# The Progression of COVID-19 and the Government Response in China

**DOI:** 10.3390/ijerph18063002

**Published:** 2021-03-15

**Authors:** Xinyi Hu, Antoine Flahault, Alexander Temerev, Liudmila Rozanova

**Affiliations:** 1 Institute of Global Studies, University of Geneva, 1205 Geneva, Switzerland; antoine.flahault@unige.ch (A.F.); alexander.temerev@unige.ch (A.T.); liudmila.rozanova@unige.ch (L.R.); 2 Institute of Global Health, University of Geneva, 1205 Geneva, Switzerland

**Keywords:** COVID-19, China, epidemic response, impact assessment

## Abstract

The ongoing pandemic of COVID-19 (Coronavirus Infectious Disease-2019) was first reported at the end of 2019 in Wuhan, China. On 30 January 2020, the WHO declared a Public Health Emergency for the novel coronavirus. On 11 March 2020, the WHO officially declared the COVID-19 outbreak as a pandemic. Due to the differences in population distribution, economic structure, degree of damage and other factors, the affected countries have introduced policies tailored to local conditions as a response to the pandemic, leading to different economic and social impacts. Considering the highly heterogeneous spreading of COVID-19 across regions, this paper takes a specific country (China) as a case study of the spread of the disease and national intervention models for the COVID-19 pandemic. The research period of this article is from 17 December to 26 April 2020, because this time period basically covered the important time nodes of the epidemic in China from animal-to-human transmission, limited human-to-human transmission, epidemic to gradual control. This study is useful for comparing the effectiveness of different interventions at various stages of epidemic development within the same country and can also promote the comparison of the epidemic response interventions of different countries. Based on the conclusions of the model simulation, this article evaluates the dual impact of the epidemic on people’s wellbeing and the economy.

## 1. Introduction

The first case of a novel coronavirus infection can be traced back to November 17, 2019 in Wuhan, Hubei Province, China. As of 26 April 2020, 68,128 cases of infection were confirmed in Hubei and a total of 83,912 cases were confirmed in China. By this time, 210 countries and territories around the world had reported COVID-19 cases.

**Figure 1:**
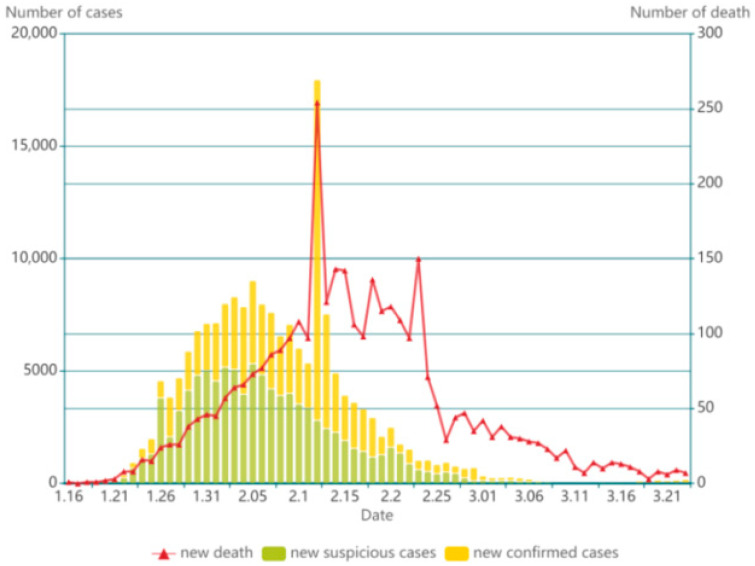
Epidemiological curve in China from 16 January to 21 March 2020.

China was the first country affected by the epidemic. In its early stages, coronavirus infections were developing rapidly in China, putting an enormous burden on the public health sector. As can be seen in Figure 1, the peak of confirmed cases and deaths appeared on 12 February 2020. The number of infections and deaths started to increase rapidly in other countries since mid-February, 2020, while the progression of China’s epidemic began to slow down by this time, showing the impact of interventions.

## 2. Objectives of Study

To study the development and intervention measures of COVID-19 in China, the first severely affected country by COVID-19, in the early stage of the epidemic is of great importance. The objective of this case study is to contextualize the progression of COVID-19 in China from multiple time dimensions: before pandemic, pandemic and post peak. First, this article will assess the risk exposure and emergency response capacity of infectious disease transmission before the pandemic, including cultural customs, demographic characteristics, medical insurance system and hospital capacity; then, this article will apply the Mass action compartmental SEIR model (S, fraction of population susceptible; E, fraction of population infected but not infective; I, fraction of population infective; R, fraction of population deceased or recovered)to simulate the epidemic progression in epicenter, Wuhan, at the same time, visualize the impact of various interventions on epidemic control to estimate its policy sensitivity; finally, this article will focus on the post-epidemic period and assess the impact of the COVID-19 both from human well-being aspects and economic aspects. The paper aims to facilitate understandings on the progression of COVID-19 in China and provide the governments with insights to optimize prevention and control plan.

## 3. Material and Method

This research is divided into six main parts: In the first part, we analyze the origin of the novel coronavirus based on the data presented by the Chinese Center for Disease Control and Prevention.

At the second part we give the demographic characteristics of China and describe the progression of the epidemiological situation (early cases). An analysis of the early distribution of cases in China could prove to be useful to further study the spread of coronavirus and develop specific interventions.

In the third part of this study, we present the general information about the China health care system; the fourth part presents some analysis of epidemic management policies and their outcomes.

In the fifth part using the SEIR (susceptible–exposed–infectious–removed) epidemiological model, we study the progress of the epidemic. Since Wuhan, as the epicenter of the epidemic, accounted for the majority of the infections in the early stage of the epidemic, the model will be divided into four stages according to the government’s adjustment of non-pharmaceutical intervention (NPI) methods for the Wuhan. To this end, model parameters, including reproduction number, fraction of susceptible exposed/day, fraction of infected infective/day and fraction of infective who recover or die/day are estimated separately for each stage and then the prediction results are aggregated.

In the last part, the paper articulates the impact of the epidemic toward people’s livelihood and state’s economy. Further interventions at the post-epidemic period should center on minimizing different impacts.

## 4. Cases Presentation

### 4.1. Origin of Infection

The exact source of the novel coronavirus and patient zero has not been confirmed. There are different versions about the origin of infection.

The first patient suffering from a novel coronavirus disease was diagnosed at Wuhan on 1 December 2019. However, there was evidence showed that that patient may not be patient number zero and the earliest infected patient can be traced back to 17 November 2019, weeks before doctors and authorities acknowledged [1,2]. However, the possibility of the existence of an infected person before this date was still not excluded.

Data on patients infected with the coronavirus were collected from Jinyintan hospitals in Wuhan from 1 to 20 January 2020. Of the 99 cases recorded at that time, 49 had contact with the Huanan Seafood Market. [3] However, subsequent research found that the Huanan seafood market probably was not the source of the infection. The researchers suggested that this site could facilitate the transmission of the virus from animal hosts to humans [4,5].

Since the outbreak, the public both inside and outside China has been investigating the possibility that the coronavirus was human-made and was deliberately or accidentally spread from the Wuhan Institute of Virology [6]. In response, the WHO stated that the available evidence indicated that the novel coronavirus originated from an animal host and was not genetically manipulated [7].

The zoonotic diseases were hypothesized to be the first 40 cases confirmed in December, 2019 [8]. An epidemiological study by the China Association for Preventive Medicine showed that the new coronavirus was most similar to the Chinese chrysanthemum bat coronavirus, associated with severe acute respiratory syndrome [9]. Consequently, many scientists believed that bats were the source of the new coronavirus.

### 4.2. Infection Spreading Characteristics and Demographics

Since 23 January 2020, the central government of China imposed a lockdown in Wuhan, individuals were not allowed to enter and leave the city without permission from authority. Nucleic acid testing was mainly for suspected symptomatic cases and their close contacts. As the epidemic was gradually controlled, the government started to allow some enterprises and social organizations that had applied for permission to resume work. From 29 March to 10 April 2020, a total of 143,056 people who had planned to return to work were screened, of which 113 were positive for nucleic acid tests, with a positive rate of 0.08%.

As of 26 April 2020, 68,128 cases of infection were confirmed in Hubei and a total of 83,912 cases were confirmed in China, in which asymptomatic infections were not counted. According to the Coronavirus Disease 2019 Prevention and Control Plan (sixth Edition) [10] issued by the National Health Commission of China, asymptomatic infection referred to those who have no clinical symptoms and those whose respiratory tract specimens are tested positive for the etiology of the new coronavirus or serum-specific IgM antibodies. Based on the definition, asymptomatic infection could be divided into two categories, one is individuals still in latent periods and the other is individuals who are asymptomatic from beginning to the end. As asymptomatic infections may be transformed into symptomatic infections in the later period and the transmission rate of asymptomatic infections is low, since the Coronavirus Disease 2019 Case Surveillance Program (Third Edition)” released on 28 January 2020 [11], the National Health Commission distinguished asymptomatic infections from suspected cases and confirmed cases. In other words, if the novel coronavirus nucleic acid test was positive but patients had no symptoms, it would not be included in the confirmed cases released every day.

**Figure 2:**
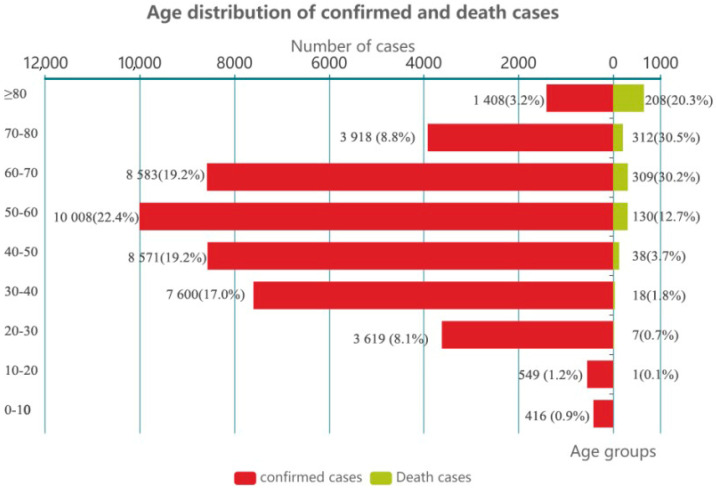
Age distribution of confirmed and death cases.

The Chinese Center for Disease Control and Prevention collected epidemiological data of all confirmed patients (44,672 cases) before 11 February 2020, 77.8% of these were between 30 to 70 years old and 81% of the deceased were older than 60 years old, as shown in Figure 2. That shows that people of all ages are at risk of infection; specifically, older people are more prone to have severe symptoms and fatal outcome compared to the younger people.

**Figure 3:**
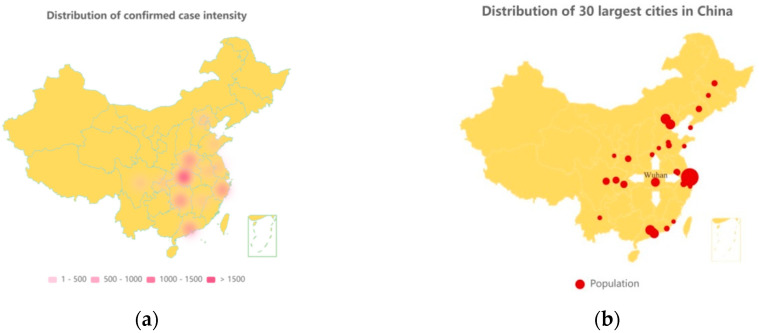
(**a**) Geographical distribution of confirmed cases; (**b**) geographical distribution of the 30 largest cities in China (Wuhan is specifically marked).

According to the National Development and Reform Commission of China, during the tourism peak from 10 January to 18 February 2020, related to the Chinese New Year holidays, there were about three billion trips [9]. Of all patients infected before 11 February 2020, approximately 30% worked as farmers, laborers, or waiters. Their mass transit was one of the factors of rapid spreading of the epidemic throughout the country. Figure 3a is a heat map of the relative density of infected population in the Chinese provinces as 23 March 2020. Figure 3b shows the country’s 30 largest cities (which are popular working places for the migrant workers) by population. Comparing the two graphs, it can be hypothesized that the spread of COVID-19 across countries is closely related to the direction of population movement.

As of 26 April 2020, Hubei province had 68,128 confirmed cases and 4512 deaths (after correction on 17 April 2020), with a mortality rate of 6.62%. The relatively low death rate in the epicenter of China compared to some countries such as Italy and Spain (the healthiest country assessed by Bloomberg in 2019 and 2020) has raised doubts from a number of researchers and the media, many have openly stated that the Chinese government is hiding the real death toll and downplays the severity of the crisis. Notably, on 17 April 2020, the official death toll in Wuhan was increased by 50% to include people who died outside hospitals and were not previously registered. China Daily noted that this statistical adjustment was based on the early stage of the outbreak in Wuhan, where testing and treatment capacity was insufficient [12,13]. Some of those infected have died without testing and treatment was not recorded as death due to COVID-19. Another reason for the adjustment is that some medical institutions were unable to effectively connect to the disease prevention and control information system at the early stage of the epidemic. The WHO’s leading COVID-19 scientist, Dr. Maria Van Kerkhove, noted China’s attempt to get closer to the real number of cases and stressed the importance of applying real numbers in epidemiological research [14]. Before 11 February 2020, as referred to the report, infected cases had direct contact with Wuhan (have been to Wuhan within 14 days of having symptoms) accounts for 85.31%. Of the 40 initially diagnosed cases in December, 2019, 27 patients had direct contact with the Huanan Seafood Market, accounting for 67.5% [15].

### 4.3. Chinese Health Care System

After decades of health care reforms, China’s basic medical system for urban and rural residents has been established. Primary medical institutions, professional public health institutions and disease prevention and control centers are set up at the city, prefecture and province levels [16].

Concerning the health insurance, a new round of medical reforms in 2009 pushed the Chinese medical system to achieve the “universal coverage” for both urban and rural residents’ medical insurance. According to the World Bank, the out-of-pocket rate in China has declined from 40.8% in 2010 to 36.05% in 2017. Since 2012, the out-of-pocket ratio keeps being lower than that of middle-income countries in average [17].

However, as it has been explained by Wang et al. [18], the Chinese public health system still faces enormous challenges. First, the mainstream thinking attaches great importance to treatment rather than prevention. Second, the current system is lacking overarching design, with insufficient capacity to respond to public health emergencies. The healthcare system is focused more on province-level hospitals as opposed to local healthcare facilities. Third, there are significant issues remain with IT and administrative support of the newly established healthcare institutions. Lastly, there is a shortage of public health personnel in China; well-trained family doctors are also in deficit. Existing healthcare personnel were already under a heavy working burden.

The healthcare needs created by the novel coronavirus pandemic have gone well beyond the capacities in many countries. Emanuel et al. [19] argued that the pandemic was likely to cause a shortage of hospital beds, ICU beds and ventilators even though the epidemic curve will flatten in an extended period. The number of hospital beds per 1000 people and the number of health care professionals per 1000 people are indicators widely used to assess the medical capacity of an area. According to data from the National Bureau of Statistics of China, in 2018, the number of hospital beds per 1000 people in China is 6.02 and the number of health care professionals per thousand people was 6.80, which were higher than those in most of the middle-income countries. However, the gap remained between China and some developed countries. For instance, Japan has 13.1 beds per thousand people and Germany had 8.00 in 2019.

During the COVID-19 outbreak, existing healthcare systems in China were under high pressure. During the initial period of government intervention, patients competed for medical resources in province-level hospitals where there were many well-trained doctors, while some other health care facilities with less trained personnel remained at surplus. People who lived in rural areas had to come to the cities to take the test, which further exacerbated the spread of the infection within the provinces. Some tests and treatments could bring heavy financial burdens for some of the affected people. Only after 23 January 2020, the Ministry of Finance and the National Healthcare Security Administration of PRC launched a series of policies to ensure that patients could get free treatment with basic medical insurance.

## 5. Epidemiological Model Simulation

### 5.1. Methodology

The susceptible—exposed—infectious—recovered (SEIR) Model has been widely applied to ascertain the spread of COVID-19 worldwide. Given that COVID-19 progression is highly sensitive to government’s non pharmaceutical interventions, the paper adopted the SEIR model and modified its parameters at each phase based on governments’ NPI at Wuhan, the epicenter of COVID-19 in China [20,21,22]. There are two reasons to adopt SEIR model in this case study: first, by dividing population into four categories, the SEIR model enables visualization of the progression of the epidemic in Hubei Province; second, research on the policy sensitivity of the SEIR model can strengthen the predictability of the development of the epidemic.

One of the major assumptions for SEIR model is that there is a homogeneous mixing of the susceptible, exposed, infected and recovered populations. The population size in a given epidemic area is constant. On 23 January 2020, the central government of China imposed a lockdown in Wuhan and other cities in Hubei province to restrict further spread of disease. From then on, all public transport, including buses, subways, railways, ferry and flights service suspended. The residents of Wuhan were not allowed to leave the cities without permission from the authorities.

Susceptible compartment ($S_{n})$ is used to represent individuals who are not yet infected, but susceptible to COVID at time n. In view of the limited spread of COVID-19 outside of Hubei province and constant population number stayed at Wuhan after 23 January 2020, the paper took Wuhan’s population of 11.08 million as the initial value of susceptible population. Exposed compartment ($E_{n}$) stands for individuals in the latent period, during which they have been infected but not yet infectious. Infectious compartment ($I_{n}$) denotes individuals who are infected and are capable to spread the disease to susceptible population. The recovered ($R_{n}$) is the compartment include people who have been removed from the disease, either due to recovery or due to death. For COVID-19, past infections does not rule out the possibility of secondary infection. In view of secondary infection being seldom reported in China due to extreme social distancing measures, the following equation (N stands for the constant number of population in Wuhan) is still feasible for tracking the progress of China’s novel coronavirus epidemic:

(1) $$S_{n}+E_{n}+I_{n}+R_{n}=N$$

The SEIR model simulates the process of the transition in the above order. At time n, individual should belong to only one of the four compartments. Baseline parameters, including reproduction number, fraction of susceptible exposed/day, fraction of infected infective/day and fraction of infective who recover or die/day, were cited from published results presented by the Chinese Center for Disease Control and Prevention [8]. The average amount of time the individual stays in each state in the first period was based on estimation from the Chinese Prevention Medicine Association: The average latent period is 5.2 days, the average interval from onset to diagnosis is 5 days, from onset to death is 9.5 days and from onset to cure is 10 days [23]. All individuals in the population have the same probability to contract the disease. Several parameters were defined to control the speed of the transition:

$R_{e}$, the effective reproduction number, stands for the average number of secondary cases per infectious case in a population. $R_{e},$ is a dynamic indicator that fluctuates sensitively with government interventions. During a period of time when the virus had no significant mutation (for instance, mutation that did not affect the transmission rate and its ability to cause disease) and official social distancing measures remain constant, $R_{e},$ was assumed to remain unchanged. ß stands for infected individual make infection-transmission contacts, which equals the number of contacts per unit time times the probability of disease transmission per contact. ε stands for rate of exposed to infective, equal to one divided by the average latent period. ƴ refers to fraction of the infected who recover a day, equal to one divided by the average duration from onset to cure. The effective reproduction number, $R_{e}$, can be calculated through the following equation:

(2) $$R_{e}=ß/ƴ$$

From compartment S to E, the total rate of infectious contact can be denoted by $ß*I_{n-1}.$ Among those infectious contacts, only a proportion S/N of those are to susceptible population, thus producing new infections. Individuals go from E to I depend on the existing exposed population (E) multiplied by the infected infective rate (ε). Individuals go from compartment I to R depend on the number of infections multiplied by the recovery rate. Thus, the number of individuals remains constant in different state at time n can be denoted by the following equations:

(3) $$S_{n}=S_{n-1}-\left(\left(S_{n-1}/N\right)*\left(ß*I_{n-1}\right)\right)$$

(4) $$E_{n}=E_{n-1}+\left(S_{n-1}/N\right)*\left(ß*I_{n-1}\right)-\left(E_{n-1}*\varepsilon \right)$$

(5) $$I_{n}=I_{n-1}+\left(E_{n-1}*\varepsilon \right)-\left(I_{n-1}*ƴ\right)$$

(6) $$R_{n}=R_{n-1}+\left(I_{n-1}*ƴ\right)$$

### 5.2. Data Collection and Parameter Values

**Figure 4:**
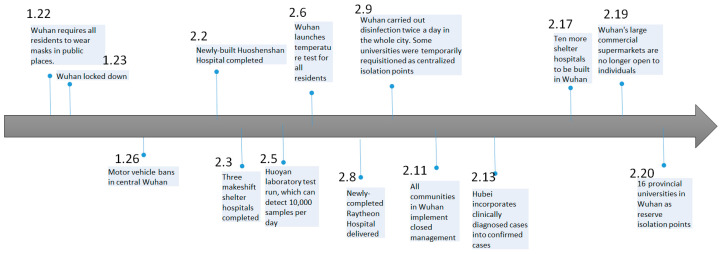
Chronology of China’s response to COVID-19.

Figure 4 shows the government’s non-pharmaceutical interventions timeline for the epidemic from 22 January to 20 February 2020, which includes extreme measures such as the establishment of a large number of temporary hospitals, complete shutdowns of commercial activities and blockades of cities. As the epidemiological map presented earlier, the government measures have a great impact on controlling the further spread of the disease.

In this regard, this article briefly divides the spread of COVID-19 in Wuhan, China into four phases:

1. Before 23 January 2020: inadequate diagnosis and awareness, lack of non-medical intervention and epidemic control and high mobility due to the Chinese New Year.
2. From 23 January to 11 February 2020: a series of compulsory closures, quarantine and surveillance measures were launched; a series of social distancing measures were implemented nationwide; the testing capacity was greatly enhanced; patients were triaged depending on the severity of the symptoms.
3. From 11 February to 22 March 2020: communities were mobilized to join the intervention efforts. All the communities in Wuhan have implemented closed management. The common practices of closed management include reducing community entrances and exits, setting up checkpoints, entering and exiting with vouchers, supervising residents to wear masks, strengthening personnel health monitoring and arranging personnel exchanges. Community staffs are obliged to deal with the needs of local residents for nucleic acid testing and medical referral.
4. From 22 March to 9 April 2020: since 22 March 2020, Hubei Province gradually loosened its 2-month lockdown. Commercial activities were steadily recovering. On 9 April 2020, Wuhan, the capital of Hubei province, lifted the lockdown.

Epidemiological data, from the beginning of the epidemic to 23 March 2020, were collected from daily reported number of cases by the Chinese Center for Disease Control in China. The initial parameter settings, including the average latent period, infection period, was directly cited from Chinese CDC [3,9]. During the model simulation process, parameters are continuously adjusted by iteratively comparing the model output with the actual confirmed infected and recovered number of cases to ensure fitness by comparing the least square. At each phase, a set of parameters with the best model fit will be selected to simulate the history of the progression of the epidemic. The division of phases is based on non-pharmaceutical interventions the government has implemented. During our study, the clinical characteristics of the novel coronavirus did not change significantly. Therefore, this article assumes that the latent period remains constant during this period (ε remains constant), while ß and ƴ are adjusted dynamically with NPIs in different phases as presented in Table 1.

### 5.3. Model Simulation

**Figure 5:**
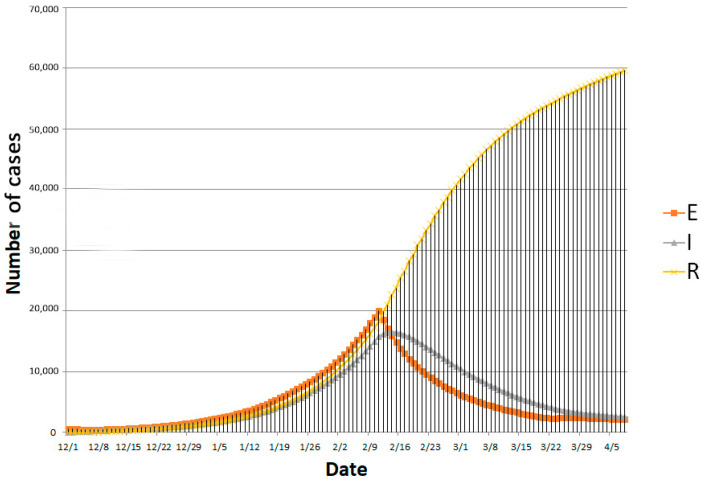
SEIR model simulation outputs from 1 December 2019, to 8 April 2020.

The SEIR model reviewed the progression of the epidemic before 23 March 2020, and also predicted the development trend of the epidemic after 23 March 2020, as can be seen in Figure 5. Noticed that government’s gradually loosening up controlling measures in phase four can increases population mobility, thereby affects the effective reproduction number. This effective reproduction number of the fourth stage is taken from the average number of selected preceding research [24,25]. At the same time, the ability to detect and cure new coronavirus infections continues to improve. It can be observed that government’s non-pharmaceutical intervention has significant impact on controlling the progression of the epidemic, but the effect could be lagged behind. Explanations for the lag effects observed in reported number of cases include limited test capacity, relatively long latent period of patients and cross-infection in isolation points and hospitals.

**Figure 6:**
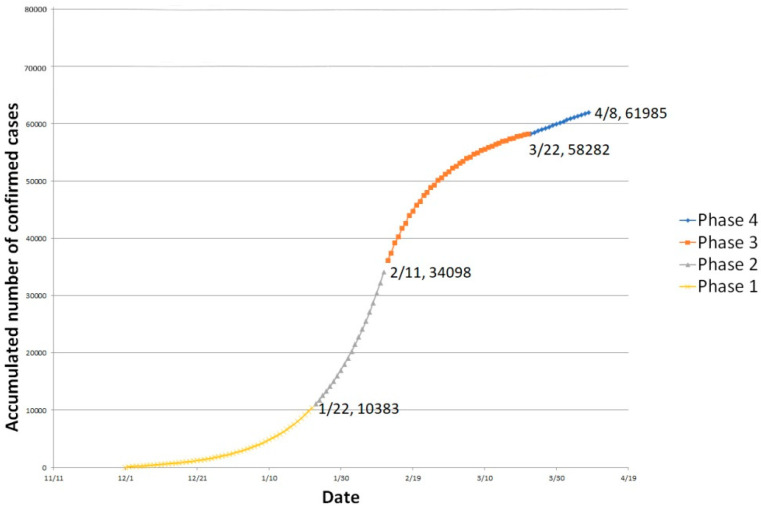
Accumulated infected cases (model outputs) from 1 December 2019, to 8 April 2020.

As shown in Figure 6, infectious cases and removed cases were combined as accumulated infected cases. The estimated number of infected cases has been shown at the end of each phase. Without considering recoveries and fatalities, the number of people infected increased at a rapid rate in the first two stages and the inflection point began to appear in the third stage after communities joined the intervention efforts. After the inflection point, the number of new infections began to slow decrease. Comparing with real time data, the model outputs ideally demonstrated the turning point and the progressive trend of the epidemic in China

### 5.4. Advantages and Limitation

Based on real data from Wuhan, the paper produced a phase-adjusted SEIR model to review the progression trend of the epidemic at Wuhan from the beginning of the epidemic to April 2020. The model overcomes the limitation that traditional models cannot dynamically consider the change of NPIs. Moreover, by matching the output of the model with the actual reported cases, the effects of relevant interventions on epidemic control in the future will be more predictable.

However, the application of the SEIR model still has some limitations. First, of all, the simulation was implemented during the first wave of COVID-19 in China. Values of the parameters in the first phase were only based on literature and data published before April 2020. Factors such as birth rate, fatality rate, test capacity and medical interventions were not taken into account. Second, the accuracy of training model through the reported number of cases is limited, especially during the first two phases when there is a large fraction of the research population were underdiagnosed patients. The reported number of cases highly depended on test capacity, supervision of suspected cases and information sharing system. Third, for COVID-19, recovered patients are still at risk of secondary infection, so they cannot be simply removed from the general population.

Although there are some limitations, the paper believed this case report could reflect a progressive domestic understanding of China’s epidemic during the first wave. It contextualizes the prevention and control of COVID-19 in China, demonstrating the value of historical research.

## 6. Impact Assessment

This public health emergency has affected all aspects of citizens’ life and commercial activities. This article will assess the impact mainly from human well-being and economic aspects.

### 6.1. Impact on Human Well-Being

First of all, a large proportion of infected population are working people, including farmers, craftsmen and service workers, many of whom are the single source of financial support for their entire extended families [8]. Secondly, a large number of infection cases were originating from local clusters, which in some cases has caused the entire communities to lose their sources of income. Furthermore, according to the notice from the Ministry of Finance and the National Healthcare Security Administration of PRC on 23 January 2020, patients who were confirmed with infection, were able to receive a free treatment. However, as there is a time lag from having symptoms to diagnosis and as the introduction of the free treatment policy took some time, many affected people were still under great financial burden for a very long period. In addition to that, a large number of cases of infection and deaths among medical personnel aggravated medical burden as well as social panic.

On the other hand, the COVID-19 outbreak also affects mental health. Duan and Zhu indicated that it is urgent to provide good care for mental health needs of the confirmed infected patients, suspected people, quarantined family members, as well as health care professionals [26]. Many people suffered from mental health problems such as depression, anxiety and stress due to the coronavirus epidemic. It is worth noting that compared to the SARS outbreak in China, a large number of online counseling services were promoted during this epidemic. In addition, online mental health education and communication activities were initiated through widely-used social media venues [27].

### 6.2. Impact on Rconomy

As Hunter, Rubin and Kim [28] predicted, the global GDP could have fallen by 12% even in the first quarter of 2020. This global pandemic has had a huge impact on the economy of every country in the world and China is no exception. The National Bureau of Statistics of China revealed that China’s total GDP has contracted by 6.8% in the first quarter of 2020 for the first time in four decades.

The suspension of a large number of economic activities in the epidemic caused a considerable impact on the private sector in the first quarter. A large number of enterprises faced debts and even went bankrupt due to the disruption of supply and capital chains. Notably, since the end of February 2020, the epidemic situation in China has gradually been brought under control. After the signal of resumption of production in March 2020, domestic demand seemed to be steadily recovering [28]. Remarkably, compensatory consumption was expected to surge after resuming commercial activities in the first quarter. Electronic markets were expected to account for a more significant portion of GDP growth after the crisis. However, considering the massive impact on economies worldwide and limited resilience of commercial activities, China might still suffer a slow-down of overall GDP growth rate in 2020 and beyond. Demand, investment and exports have long been considered as the troika that drives economic growth in China. Therefore, this chapter will assess the economic impact based on these three areas.

#### 6.2.1. Consumption

The first hit of pandemic on economy is a sizable decline in aggregate supply and demand. The total retail sales of consumer goods is the total amount of consumer goods sold directly to urban and rural residents and social groups in various sectors of the national economy, which can be a strong indicators reflecting the level of economic activities during a period of time. According to data from the National Bureau of Statistics of China showed that, from January to April, 2020, the total retail sales of consumer goods decreased significantly year-on-year. In March and April, 2020, the negative growth reached −15.8% and −7.5%, respectively. The lockdown in an effort to stop the spread of the pandemic has restricted people from buying goods offline and the suspension of domestic and international express delivery services has brought online consumption to a halt.

From the perspective of foreign trade, imports and exports have declined in varying degrees from January to April, 2020, year-on-year. Specifically, the decline in exports in January and February, 2020, was greater than that in imports due to rapidly shrinking production volume and some governments’ distrust of the safety of products imported from China. According to the National Bureau of Statistics of China, the decline in imports in March and April, 2020, was greater than that in exports due to the gradual recovery of domestic productivity and weakened foreign productivity due to the epidemic. Apart from goods trade, cross-broader merchandise and service trade almost stopped during the first wave of COVID-19 in China, mainly due to social-distancing measures issued by different states. All in all, the pandemic has significantly reduced the consumption, especially consumers’ demand on non-essential goods and services [29]. The market share of import and export also fluctuates sharply. China’s surplus in international trade remains unchanged.

The Consumer Price Index (CPI) is a measure that examines the weighted average price of a basket of goods and services. CPI was largely used to evaluate the price levels and purchasing power of citizens. As for the COVID-19 pandemic, the gradual blockade from cities to countries worldwide puts pressure on the supply of domestic and imported goods and services. As a response, the Chinese General Administration of Market Supervision issued strict guidance on 7 February 2020. The document stipulated that the prices of personal protective equipment (PPE) and basic livelihood commodities should be kept within a certain range. To this end, specific solutions include ensuring the producers’ output, suppliers’ cross-regional allocating resources in a timely manner and retailers’ guaranteeing their stock of goods have been brought into force. Generally, despite inflationary pressures, the government’s timely responses have effectively brought down the price volatility. In January and February, 2020, the CPI rose 5.4% and 5.2% year-on-year. Demand on essential goods increased, while on non-essential goods decreased as people incline to save for emergency. Notably, the price of a basket of food, tobacco and alcohol products increased significantly, with CPI rising 15.2% and 16.0%, respectively. With the implementation of a series of policies, the CPI in March and April, 2020, was gradually brought into control, with overall CPI rise 4.3% and 3.3%. In summary, the increase in CPI was significantly higher than that of previous years, among which, the price of necessities led the rise. Governmental policies on material supply and allocation, as well as regulation on price brought the price within control. The gradually stable price of necessities during epidemic can reflect citizen’s confidences in the economy.

#### 6.2.2. Investments

The uncertainty induced by the pandemic has driven investors to hold back and postpone their decisions on investment. As the epidemic spreads around the world, capital in the investment market is shrinking.

From the perspective of investors, on the one hand, financial institutions were operated under great risk. Take the example of banks as an example; banks could face declined general deposits and increased credit and default risk, with lower expectation on portfolio investment returns. Insurance companies could face increased claims and lower premiums to pay due to people’s lower affordability [29]. On the other hand, companies may be forced to suspend their work due to the inability to mobilize employees and purchase materials. In the absence of sustained profits, these companies may break their capital chain due to fixed asset expenditures, employee salaries, failure to apply for bank loans and other reasons. With the increase in credit risk, a large number of small and medium-sized companies were forced to lay-off employees to keep their financial sustainability, which would significantly increase the unemployment rate, thereby further exacerbate individual default risk. To respond to this threat, China’s Ministry of Finance introduced a series of measures to support small and micro enterprises to resume production activities, including reductions of tax burden and corporate financing costs [30].

From the perspective of underlying assets, bonds and gold that were considered to be able to hedge risks were sold on a large scale without discrimination during the epidemic. As a result, bond yields fell sharply, with gold prices continuing to rise [31,32,33]. As for oil, the epidemic directly affects oil demand and has a greater impact on related industries. Although the financial sector has no direct association with oil, it can be sensitive to oil price risk due to their lending and portfolio management [34]. Due to the co-movement of oil price risk and industrial stock returns, oil price risk has been regarded as an important indicator to evaluate and predict the trend of economy. The sharp drop in oil prices during the epidemic reflects the pessimistic expectations of the global economy. Admittedly, in the short term, the sharp drop in oil prices can save residents’ transportation costs, promote consumption and reduce China’s oil import costs and linked industry operating costs. However, in the long term, oil price shocks may promote the introduction of competition mechanisms, creating deflation and ultimately, aggravating the economy of oscillation.

PMI (purchasing managers index) is widely used to measure the general performance of the Chinese economy. It measures the status of the industries in all aspects of production through monthly surveys of private sector companies, which can also indicate the potential investment confidence of these companies. In addition, it has long been regarded as the leading indicator of PPI (producer price index) and CPI. In February, 2020, there was a surge in the number of confirmed COVID-19 cases and deaths in China; simultaneously, the government has further strengthened its grip by limiting the production and selling of commodities nationwide. Corresponding to that trend, the overall PMI indexes (including manufacturing and non-manufacturing department) in February, 2020, fell to 28.9% (far below the threshold of 50.0%), indicating a substantial decline in production. Remarkably, data for March and April, 2020, show a sharp rebound in market confidence, with overall PMI picked up by 53.0% and 53.4%, respectively. Market confidence has gradually recovered following the prevention and control of the epidemic. However, even though it has been expected that China has already gone through the most severe phase of the COVID-19 outbreak, the downturn in the world economy may additionally affect the availability of new investments.

#### 6.2.3. Export

According to data from the National Bureau of Statistics of China, from December, 2019 to February, 2020, China’s export volume has decreased by 17.2%. Imports and exports together declined by 11%, among which the decline significantly affected export products’ competitivity, such as electromechanical products, mobile phones and labor-intensive products like toys and furniture. The extended Spring Festival holiday and the spread of the epidemic in China in the first two months of 2020 prevented many export-oriented companies from fulfilling overseas orders as scheduled. Although the government has issued a signal to resume all work in March, 2020, the rebound of China export may still be limited due to the aggravation of pandemic worldwide. After the resumption of work, the export volume still underperformed in March, 2020, compared with previous years, with a 6.6% decline in the total export volume. The impact of the epidemic on China’s exports is profound, because on the one hand, it takes time for China to restore its original productivity; on the other hand, the spread of the world epidemic has shrunk foreign imports.

In terms of the US–China trade war, even though at the beginning of 2020, China and the United States signed a trade deal aiming to end the 2-year economic conflict, the pandemic may still dampen the China–US trade volume by the residual shrinking of import and export. Although the pandemic has severely affected both economies, the China–US trade war is not expected to stop in the near future [35,36]. That means that Chinese and American companies need to maintain sufficient business flexibility and further adjust their business scope following the development of the epidemic and the trade war. Moreover, the China–US relationship was also compromised by a technological rivalry and the lack of cooperation in addressing the pandemic.

## 7. Conclusions

The COVID-19 pandemic began in December 2019 and has caused numerous hospitalizations and deaths worldwide. As the first country to report the virus, China suffered severe setbacks due to its dense population, frequent internal travel and limited knowledge on how to handle a major healthcare crisis in the early stages of the epidemic.

The Chinese Government have launched a series of response measures since late January, 2020, which have had a significant effect on reducing the epidemic spread, and in particular, non-pharmaceutical interventions. Additionally, medical interventions and communities’ involvement significantly improved the case management practices both for treatments and rehabilitation. Presently, the epidemic situation in China has essentially been brought under control. Undoubtedly, the pandemic has severely threatened human wellbeing and the economy in China and elsewhere. However, with the release of the signal to restart production at the end of March, 2020, domestic supply and demand have started to pick up. Concerning the Chinese GDP performance, the economic rebound is still limited by domestic and foreign economic conditions, despite financial and monetary policy support from the Chinese government. During the post-epidemic era, further interventions to prevent and control the epidemic and maintain the economy should consider the impact each intervention will bring.

## Figures and Tables

**Table 1:** Parameters in different phases.

Phase	ß	ε	ƴ	$R_{e}$
Phase 1	0.22	0.19	0.10	2.20
Phase 2	0.20	0.19	0.12	1.67
Phase 3	0.095	0.19	0.12	0.79
Phase 4	0.114	0.19	0.13	0.88

## Data Availability

Not applicable.
